# Evaluation of oral fluralaner (Bravecto^®^) for efficacy against nymphs of *Amblyomma americanum* and *Rhipicephalus sanguineus* (*sensu lato*)

**DOI:** 10.1186/s13071-020-04179-y

**Published:** 2020-06-18

**Authors:** Kelly Allen, Susan Little, Melissa Petersen, Jeff Gruntmeir, Anne Barrett, Brian Herrin, Lindsay Starkey, Fangshi Sun, Frank Guerino

**Affiliations:** 1 Department of Veterinary Pathobiology, Oklahoma State University, College of Veterinary Medicine, Stillwater, Oklahoma 74078 USA; 2 Merck Animal Health, De Soto, Kansas 66018 USA; 3 Department of Comparative Diagnostics and Population Medicine, University of Florida, College of Veterinary Medicine, Gainesville, Florida 32611 USA; 4 Merck Animal Health, Madison, New Jersey 07940 USA; 5 Diagnostic Medicine/Pathobiology, Kansas State University, College of Veterinary Medicine, Manhattan, Kansas 66506 USA; 6 Department of Pathobiology, Auburn University, College of Veterinary Medicine, Auburn, Alabama 36849 USA

**Keywords:** *Amblyomma americanum*, Brown dog tick, Infestation, Isoxazoline, Lone star tick, *Rhipicephalus sanguineus* (*sensu lato*)

## Abstract

**Background:**

*Amblyomma americanum* and *Rhipicephalus sanguineus* (*sensu lato*) nymphs commonly feed on and transmit pathogens to dogs (*Canis familiaris*). Control of immature and adult tick life stages is necessary to fully protect animals. We evaluated efficacy of oral fluralaner (Bravecto^®^) against induced infestations with *A. americanum* and *R. sanguineus* (*s.l*.) nymphs on dogs in two experiments.

**Methods:**

In each experiment, 10 dogs were administered oral fluralaner chewable tablets one time on Day 0 at a targeted minimum dose of 25 mg/kg body weight and 10 dogs remained non-treated controls. Dogs were infested with two groups of 50 *A. americanum* nymphs and two groups of 50 *R. sanguineus* (*s.l*.) nymphs on Days -1, 6, 28, 56 and 84. At 48 h and 72 h post-infestation, nymphs were collected from dogs, assessed as live or dead, and enumerated into categories defining attachment and engorgement status. Fluralaner efficacy was determined in separate analyses against all live nymphs and against live-fed nymphs, i.e. live nymphs that were attached to dogs at the time of collection and/or were engorged. Fluralaner was considered effective when mean numbers of live ticks were reduced in fluralaner-treated dogs by ≥ 90%.

**Results:**

Fluralaner efficacy against all live and live-fed *A. americanum* nymphs in the first experiment was > 94% on all collection days. Efficacy against all live *R. sanguineus* (*s.l*.) nymphs in the first experiment was  > 96% on all collection days  excluding the 48 h counts for infestations on Days 28 (83.7%), 56 (82.9%) and 84 (86.7%); efficacy against live-fed *R. sanguineus* (*s.l*.) nymphs was > 95% on all 48 h/72 h count days. Fluralaner efficacy against all live *A. americanum* nymphs in the second experiment was > 93% on all collection days for 8 weeks excluding the 48 h count for infestation on Day 56 (87.8%); efficacy against live-fed *A. americanum* nymphs was > 91% on all count days for 8 weeks. Efficacy against all live *R. sanguineus* (*s.l*.) nymphs in the  second experiment was > 91% on all 72 h collection days  except for infestations on Days 28 (76.8%) and 56 (86.3%); efficacy against live-fed *R. sanguineus* (*s.l*.) nymphs was 100% on all 72 h count days.

**Conclusions:**

A single administration of oral fluralaner to dogs is effective against *A. americanum* and *R. sanguineus* (*s.l*.) nymphs for up to 12 weeks.
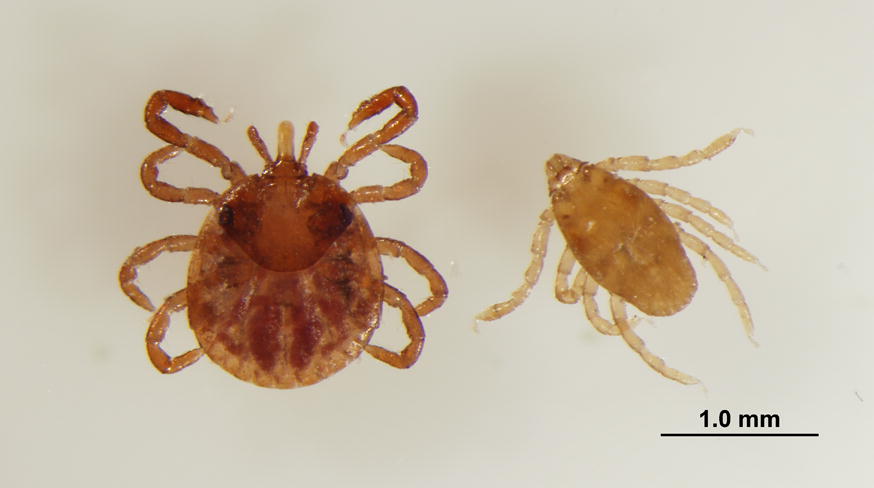

## Background

Deleterious effects of tick infestations on animals are numerous. Direct consequences as a result of tick feeding include anemia, dermal irritation, hypersensitivity, and toxicosis. Also, a variety of pathogenic agents are transmitted to animals by ticks during blood-meal acquisition, which may result in illness and death in infested hosts [1–3]. Control of immature stages as well as adults is important for complete protection of animals against tick infestations.

A tick species that feeds on a variety of animals and is well-documented on dogs (*Canis familiaris*) during larval, nymphal, and adult stages is *Amblyomma americanum*, the lone star tick [4–6]*. Amblyomma americanum* are known to transmit numerous infectious agents as nymphs and adults to dogs and humans including *Ehrlichia chaffeensis* and *Ehrlichia ewingii* [5–8]. Long suspected as a vector of *Rickettsia rickettsii*, *A. americanum* was recently experimentally demonstrated to transmit this pathogen to dogs as both nymphal and adult stages [9, 10]. *Rhipicephalus sanguineus* (*sensu lato*), commonly called brown dog ticks, are found worldwide and all instars preferentially feed on dogs [11]. *Rhipicephalus sanguineus* (*s.l*.) nymphs and adults transmit *Anaplasma platys*, *Babesia* spp., *Ehrlichia canis*, *Hepatozoon canis* and *R. rickettsii* [4, 11–16]. Infected *R. sanguineus* (*s.l*.) nymphs, in particular, were implicated in a *R. rickettsii* outbreak in Arizona in 2003 involving human fatalities; experimental data confirmed vector competence of immature stages of *R. sanguineus* (*s.l*.) [17–19].

Control of immature ticks on dogs as well as adult ticks is important in reducing infestations, decreasing risk of pathogen transmission, and limiting tick populations in the environment. *In vivo* trials determining acaricidal compound efficacy against immature *A. americanum* and *R. sanguineus* (*s.l*.) are lacking. The isoxazoline fluralaner is a systemic compound that exerts acaricidal and insecticidal activity by blocking gamma-aminobutyric acid (GABA)- and glutamate-gated chloride channels [20]. Oral fluralaner (Bravecto^®^) is indicated for the treatment and control of tick infestations on dogs, including *A. americanum* for 8 weeks and *R. sanguineus* (*s.l*.) for 12 weeks, as demonstrated using adult ticks in both laboratory experiments and field trials [20, 21]. Here, we present the results of two Good Clinical Practice (GCP) laboratory experiments evaluating the efficacy of oral fluralaner against *A. americanum* and *R. sanguineus* (*s.l*.) nymphs for 12 weeks after administration of the minimum commercial dose (25 mg/kg body weight).

## Methods

The efficacy of 13.64% w/w fluralaner chewable tablets (Bravecto^®^, Merck Animal Health, Madison, New Jersey) against *A. americanum* and *R. sanguineus* (*s.l*.) nymphs was evaluated in two separate randomized, blinded laboratory experiments with parallel-group designs. Each experiment was conducted in compliance with Good Clinical Practice (GCP) guidelines (VICH GL9) [22] and the World Association for the Advancement of Veterinary Parasitology (WAAVP) guidelines for evaluating parasiticide efficacy against flea and tick infestations on cats and dogs [23]. The duplicate experiments were conducted to document fluralaner efficacy against different genetic isolates of nymphal *A. americanum* and *R. sanguineus* (*s.l*.) to demonstrate the compound’s utility in the field against immatures of the species.

Twenty laboratory reared neutered male or intact female beagles, each at ≥ 6 months of age, were used in the two experiments. To reduce the total number of animals used between both experiments, 8 non-treated control dogs from the first experiment were transferred to the second experiment and re-used in control or treated groups along with 12 newly acquired, non-treated dogs. The dogs were initially weighed and physically examined to ensure overall health, and allowed an acclimation period of at least 28 days prior to experiment commencement. Dogs were housed individually or in pairs inside raised kennels with plastic-coated expanded metal floors in designated climate-controlled rooms provided a 12:12 h L:D cycle.

*Amblyomma americanum* were obtained from colonies maintained by Oklahoma State University (OSU) (Stillwater, Oklahoma) and Texas A&M University (College Station, Texas) and *R. sanguineus* (*s.l*.) were from colonies maintained by OSU and El-Labs (Soquel, California) [24]. In both experiments, dogs were infested with 50 *R. sanguineus* (*s.l*.) nymphs on Day-28 for treatment group allocation. For all infestations, the hair on the lateral thorax was clipped and nymphs were applied directly to shaved skin beneath bandage pads (Band-Aid^®^, Johnson & Johnson, New Brunswick, New Jersey, USA). Cotton stockinette (Stockinette, Medline, Mundelein, Illinois, USA) and elastic bandage wrap (Elastikon^®^, Johnson & Johnson, New Brunswick, New Jersey, USA) was used to further contain nymphs and facilitate later recovery and accurate counting. Elizabethan collars (Well & Good E-Collar, Petco, San Diego, California, USA) were placed on dogs as needed to prevent removal of the tick containment materials. When infested with ticks, dogs were housed individually in kennels. After each counting event, we discarded all ticks.

*Rhipicephalus sanguineus* (*s.l*.) nymphs applied on Day-28 were removed after 72 ± 4 h (Day-25) and enumerated into the following 8 categories indicating vitality, attachment, and engorgement status: live, attached and engorged; live, attached and not engorged; live, free and engorged; live, free and not engorged; dead, attached and engorged; dead, attached and not engorged; dead, free and engorged; dead, free and not engorged. On all sampling days, nymphs were collected using fine forceps and examined using a stereomicroscope. Live status of ticks was determined by movement of legs. If no movement was observed, nymphs were gently probed with forceps and stimulated with CO_2_ (exhaled air) to assess leg movement. Any tick exhibiting leg movement at the time of removal was considered as live. Nymphs were considered attached if mouthparts were partially to completely anchored within the canine dermis at the time of removal. Engorgement status of ticks was determined by inspecting the ventral idiosoma; nymphs that had ingested blood appeared distended and light brown in color while nymphs that had not fed were completely flat and dark in color. Numbers of live and dead nymphs were recorded according to attachment and engorgement status.

Dogs were ranked according to *R. sanguineus* (*s.l.*) live-fed nymph counts (i.e. live nymphs categorized as attached and engorged, attached and not engorged, and free and engorged) and animal identification number, then randomly assigned to treatment and control groups. After treatment group allocation, dogs were allowed to co-mingle with the other dogs of the same group. Observations and procedures after animals were randomized, including infestations, tick counts, and tick assessments, were performed by individuals masked to dog treatment status. Sterile gloves, surgical gowns, and shoe-covers were worn when treating and mock-treating dogs, infesting dogs with ticks, and collecting ticks from infested dogs to prevent product cross-contamination between animals. Experiment participants and personnel responsible for husbandry changed protective wear after handling each dog.

Dogs were treated or mock-treated on Day 0 according to assigned group. The number and size of 13.64% w/w fluralaner flavored chewable tablets was determined for each dog by body weight and the targeted minimum dose of 25 mg/kg. Animals were offered a small amount of food (~1/2 cup) and allowed 20 min to eat before treatment. The fluralaner dose was placed in the back of the dog’s oral cavity over the tongue to initiate swallowing. Animals were monitored for 1 h after fluralaner administration for adverse reactions and to ensure that doses were retained.

To evaluate fluralaner efficacy against nymphal *A. americanum* and *R. sanguineus* (*s.l*.), dogs were infested 20 ± 2 h prior to and 1 week (Day 6), 4 weeks (Day 28), 8 weeks (Day 56), and 12 weeks (Day 84) after treatment administration. At infestation, each dog was challenged with two groups of 50 *A. americanum* nymphs and two groups of 50 *R. sanguineus* (*s.l*.) nymphs unless otherwise stated in results. Groups of ticks were applied to separate caudal and cranial locations along the lateral thoraco-abdominal areas of dogs: left side (*A. americanum*); right side (*R. sanguineus* (*s.l*.)). Nymphs were collected from caudal locations 48 h after infestation and from cranial locations 72 h after infestation. At removal, nymphs were morphologically confirmed as *A. americanum* or *R. sanguineus* (*s.l*.) and enumerated into the aforementioned categories delineating vitality, attachment, and engorgement status.

### Fluralaner efficacy assessment

SAS version 9.3 was used as the primary software for data analyses [21, 25]. Evaluations of fluralaner efficacy against each tick species were considered valid if the number of all live nymphs (attached and engorged, attached and not engorged, free and engorged, and free and not engorged) removed from individual dogs at each collection time was at least 12 ticks on at least 6 control dogs. The log-counts of all live nymphs from treated dogs were compared to the log-counts of all live nymphs from control dogs at each 48 h and 72 h post-infestation collection time. Additionally, the log-counts of live-fed nymphs (live nymphs that were attached to dogs at the time of collection and/or were engorged) from treated dogs were compared to live-fed nymphs from control dogs at each 48 h and 72 h post-infestation evaluation time. Enumerated live-fed nymphs were categorized as attached and engorged, attached and not engorged, and free and engorged (i.e. free and not engorged counts were excluded from the fluralaner efficacy evaluation). A linear mixed model which included treatment group as a fixed effect and block as a random effect was used for comparisons. In case of any zero count, the logarithm of (count + 1) transformation was applied to all observations prior to analysis [22, 25].

For both tick species, geometric means of all live nymphs and live-fed nymphs from each count day were calculated; t-tests were performed (*P *< 0.05). Fluralaner control efficacy (%) was calculated against all live and live-fed nymphs at both post-infestation collection times (48 h and 72 h) using Abbott’s formula [23]:

$${\text{Efficacy}}\;(\% ) = {\text{100 X }}({\text{M}}_{{\text{c}}} - {\text{M}}_{{\text{T}}} )/{\text{M}}_{{\text{c}}}$$

where M_c_ is the mean (geometric) live nymph count from the control group and M_T_ is the mean (geometric) live nymph count from the fluralaner-treated group. Fluralaner efficacy against *A. americanum* and *R. sanguineus* (*s.l*.) nymphs was declared when live counts from treated dogs were reduced by ≥ 90% [23].

## Results

All dogs accepted fluralaner readily and retained treatments in both experiments. Based on body weight at treatment, each dog received a single dose of 26–36 mg/kg fluralaner. No adverse events associated with treatment were observed. Two non-treated dogs in the first experiment developed localized inflammatory reactions associated with *A. americanum* attachment on Day 28; lesions on one dog resolved rapidly, but one dog had persistent dermal inflammation and was not infested with *A. americanum* on Days 56 and 84. In the second experiment, one dog developed a localized, inflammatory lesion associated with *R. sanguineus* (*s.l.*) attachment on Day-28 (prior to fluralaner administration). This dog was not infested with *R. sanguineus* (*s.l.*) at the caudal location (for 48 h count) on Days -1 and 7, but was infested at this location on subsequent dates.

Adequacy of infestation with *A. americanum* nymphs (at least 12 live ticks on at least 6 non-treated dogs) was achieved in both experiments for all 48 h and 72 h counts. Adequacy of infestation with *R. sanguineus* (*s.l*.) nymphs was achieved in the first experiment for all 48 h and 72 h counts and for all 72 h counts in the second experiment. Due in part to a bandage breach on some dogs, 48 h *R. sanguineus* (*s.l*.) nymph infestations were not adequate in the second experiment and these data were not included or interpreted. When live-fed nymphs were assessed, adequacy of infestation was not achieved for *R. sanguineus* (*s.l*.) in the first experiment at the 48 h count on infestation Day 56, and in the second experiment at 72 h counts on infestation Days 28, 56 and 84. Because infestation adequacy was achieved for all live *R. sanguineus* (*s.l*.) nymphs on these specific count days however, the corresponding live-fed nymph efficacy data are also reported.

Geometric means of *A. americanum* and *R. sanguineus* (*s.l*.) all live (L) nymph counts and live-fed (LF) nymph counts, statistical results, and calculated fluralaner efficacies (%) are presented in Tables 1, 2, 3, 4.

**Table 1:** Percent efficacy of fluralaner based on all live and live-fed *Amblyomma americanum* nymphs from control and treated dogs (Experiment 1). Each group had 10 dogs unless otherwise noted

Infestation day	Count day	Control		Fluralaner						Efficacy	
		GM (L)	GM (LF)	GM (L)	GM (LF)	*t*-value (L)	*t*-value (LF)	*P*-value (L)	*P*-value (LF)	% (L)	% (LF)
− 1	1	43.9	42.6	1.2	0.0	*t*_(18.0)_ = 10.5	*t*_(18.0)_ = 165.4	< 0.001	< 0.001	97.3	100
	2	41.8	41.8	0.2	0.2	*t*_(18.0)_ = 27.9	*t*_(18.0)_ = 27.9	< 0.001	< 0.001	99.5	99.5
6	8	47.0	45.3	0.8	0.3	*t*_(9.0)_ = 16.8	*t*_(18.0)_ = 21.3	< 0.001	< 0.001	98.4	99.4
	9	46.9	46.9	0.8	0.5	*t*_(18.0)_ = 18.3	*t*_(18.0)_ = 18.8	< 0.001	< 0.001	98.2	98.9
28	30	47.5	45.6	0.7	0.6	*t*_(9.0)_ = 10.8	*t*_(9.0)_ = 11.2	< 0.001	< 0.001	98.5	98.6
	31	43.9	43.9	0.7	0.7	*t*_(18.0)_ = 12.9	*t*_(18.0)_ = 12.9	< 0.001	< 0.001	98.4	98.4
56	58	46.0 ^a^	46.0^a^	1.6	1.6	*t*_(8.8)_ = 8.7	*t*_(8.8)_ = 8.7	< 0.001	< 0.001	96.5	96.5
	59	46.2 ^a^	46.2^a^	0.3	0.3	*t*_(8.9)_ = 18.9	*t*_(8.9)_ = 18.9	< 0.001	< 0.001	99.3	99.3
84	86	43.1^a^	42.4^a^	2.3	2.0	*t*_(9.2)_ = 5.5	*t*_(9.2)_ = 5.9	< 0.001	< 0.001	94.7	95.2
	87	43.9 ^a^	43.9^a^	1.1	1.1	*t*_(17.0)_ = 6.7	*t*_(17.0)_ = 6.7	< 0.001	< 0.001	97.6	97.6

^a^Only nine dogs available for infestation due to one dog having inflammatory tick attachment site reactions

*Abbreviations*: GM, geometric mean; L, all live (attached and engorged, attached and not engorged, free and engorged, and free and not engorged); LF, live-fed (attached and engorged, attached and not engorged, and free and engorged)

**Table 2:** Percent efficacy of fluralaner based on all live and live-fed *Rhipicephalus sanguineus* (*sensu lato*) nymphs from control and treated dogs (Experiment 1). Each group had 10 dogs

Infestation day	Count day	Control		Fluralaner						Efficacy	
		GM (L)	GM (LF)	GM (L)	GM (LF)	*t*-value (L)	*t*-value (LF)	*P*-value (L)	*P*-value (LF)	% (L)	% (LF)
− 1	1	37.3	25.6	0.9	0.1	*t*_(18.0)_ = 12.8	*t*_(18.0)_ = 26.8	< 0.001	< 0.001	97.7	99.7
	2	30.1	29.8	0.1	0.0	*t*_(9.0)_ = 28.0	*t*_(18.0)_ = 30.5	< 0.001	< 0.001	99.8	100
6	8	28.1	25.7	1.0	0.0	*t*_(18.0)_ = 9.0	*t*_(18.0)_ = 29.9	< 0.001	< 0.001	96.4	100
	9	29.7	29.7	0.0	0.0	*t*_(9.0)_ = 41.0	*t*_(18.0)_ = 41.0	< 0.001	< 0.001	100	100
28	30	15.5	12.6	2.5	0.0	*t*_(9.0)_ = 5.8	*t*_(18.0)_ = 14.7	< 0.001	< 0.001	83.7	100
	31	21.4	20.4	0.1	0.0	*t*_(9.0)_ = 20.4	*t*_(18.0)_ = 21.9	< 0.001	< 0.001	99.3	100
56	58	13.6	10.0	2.3	0.0	*t*_(9.0)_ = 6.6	*t*_(18.0)_ = 13.6	< 0.001	< 0.001	82.9	100
	59	21.7	21.6	0.2	0.0	*t*_(9.0)_ = 18.1	*t*_(18.0)_ = 23.8	< 0.001	< 0.001	99.1	100
84	86	19.9	16.3	2.6	0.7	*t*_(9.0)_ = 6.2	*t*_(9.0)_ = 7.0	< 0.001	< 0.001	86.7	95.7
	87	24.7	23.8	0.9	0.6	*t*_(9.0)_ = 7.5	*t*_(9.0)_ = 7.5	< 0.001	< 0.001	96.3	97.5

*Abbreviations*: GM, geometric mean; L, all live (attached and engorged, attached and not engorged, free and engorged, and free and not engorged); LF, live-fed (attached and engorged, attached and not engorged, and free and engorged)

**Table 3:** Percent efficacy of fluralaner based on all live and live-fed *Amblyomma americanum* nymphs from control and treated dogs (Experiment 2). Each group had 10 dogs

Infestation day	Count day	Control		Fluralaner						Efficacy	
		GM (L)	GM (LF)	GM (L)	GM (LF)	*t*-value (L)	*t*-value (LF)	*P*-value (L)	*P*-value (LF)	% (L)	% (LF)
− 1	1	41.3	41.3	2.1	1.6	*t*_(18.0)_ = 9.8	*t*_(18.0)_ = 10.3	< 0.001	< 0.001	94.9	96.2
	2	40.8	40.8	1.2	1.2	*t*_(9.0)_ = 11.2	*t*_(9.0)_ = 11.2	< 0.001	< 0.001	97.1	97.1
6	8	40.5	40.2	0.8	0.5	*t*_(18.0)_ = 14.7	*t*_(18.0)_ = 17.6	< 0.001	< 0.001	98.1	98.7
	9	45.7	45.7	0.6	0.6	*t*_(9.0)_ = 16.0	*t*_(9.0)_ = 16.0	< 0.001	< 0.001	98.7	98.7
28	30	43.0	42.0	0.8	0.6	*t*_(18.0)_ = 14.2	*t*_(18.0)_ = 16.5	< 0.001	< 0.001	98.1	98.6
	31	42.5	42.4	0.4	0.3	*t*_(9.0)_ = 14.9	*t*_(18.0)_ = 16.1	< 0.001	< 0.001	99.0	99.2
56	58	41.4	41.0	5.1	3.4	*t*_(9.0)_ = 5.7	*t*_(9.0)_ = 5.5	< 0.001	< 0.001	87.8	91.6
	59	37.2	36.9	2.4	2.3	*t*_(18.0)_ = 5.6	*t*_(18.0)_ = 5.7	< 0.001	< 0.001	93.5	93.9
84	86	37.2	37.0	11.8	10.9	*t*_(18.0)_ = 2.6	*t*_(18.0)_ = 2.6	0.019	0.019	68.3	70.6
	87	35.6	35.5	7.8	7.8	*t*_(9.0)_ = 2.9	*t*_(9.0)_ = 2.9	0.018	0.019	78.0	78.0

*Abbreviations*: GM, geometric mean; L, all live (attached and engorged, attached and not engorged, free and engorged, and free and not engorged); LF, live-fed (attached and engorged, attached and not engorged, and free and engorged)

**Table 4:** Percent efficacy of fluralaner based on all live and live-fed *Rhipicephalus sanguineus* (*sensu lato*) nymphs from control and treated dogs (Experiment 2). Each group had 10 dogs

Infestation day	Count^a^ day	Control		Fluralaner						Efficacy	
		GM (L)	GM (LF)	GM (L)	GM (LF)	*t*-value (L)	*t*-value (LF)	*P*-value (L)	*P*-value (LF)	% (L)	% (LF)
− 1	2	21.8	20.6	0.0	0.0	*t*_(9.0)_ = 39.1	*t*_(18.0-)_ = 39.0	< 0.001	< 0.001	100	100
6	9	13.4	11.9	0.8	0.0	*t*_(9.0)_ = 7.6	*t*_(18.0)_ = 10.8	< 0.001	< 0.001	94.0	100
28	31	10.0	7.5	2.3	0.0	*t*_(9.0)_ = 4.3	*t*_(18.0)_ = = 11.1	0.002	< 0.001	76.8	100
56	59	13.0	9.8	1.8	0.0	*t*_(18.0)_ = 6.9	*t*_(18.0)_ = 14.3	< 0.001	< 0.001	86.3	100
84	87	13.0	10.4	1.0	0.0	*t*_(18.0)_ = 7.1	*t*_(18.0)_ = 21.4	< 0.001	< 0.001	91.9	100

*Abbreviations*: GM, geometric mean; L, all live (attached and engorged, attached and not engorged, free and engorged, and free and not engorged); LF, live-fed (attached and engorged, attached and not engorged, and free and engorged)

^a^48 h count data (Days 1, 8, 30, 58 and 86) are not included because adequacy of infestations on control dogs were not achieved

### Experiment 1

All live and live-fed *A. americanum* nymph counts from fluralaner-treated dogs at 48 h and 72 h post-infestation were significantly lower (*P *< 0.001) than counts from control dogs for all tick challenge days. Fluralaner efficacy against all live and live-fed *A. americanum* nymphs was > 94% at 48 h and 72 h for all time points evaluated through infestation Day 84 (Table 1).

All live and live-fed *R. sanguineus* (*s.l*.) nymph counts from fluralaner-treated dogs at 48 h and 72 h post-infestation were significantly lower (*P *< 0.001) than counts from control dogs for all tick challenge days. Fluralaner efficacy against all live *R. sanguineus* (*s.l*.) nymphs at 48 h was variable and ranged from 82.9% to 97.7%, but was > 96% for all 72 h counts; fluralaner efficacy against live-fed *R. sanguineus* (*s.l*.) nymphs was > 95% at 48 h and 72 h counts for all tick challenge days through infestation Day 84 (Table 2).

### Experiment 2

All live and live-fed *A. americanum* nymph counts from fluralaner treated dogs at 48 h and 72 h post-infestation were significantly lower (*P *≤ 0.018) than counts from control dogs for all tick challenge days. Fluralaner efficacy against all live *A. americanum* nymphs was > 94% at 48 h and 72 h counts through infestation Day 28, was 87.8% (48 h) and 93.5% (72 h) on infestation Day 56, and was 68.3% (48 h) and 78.0% (72 h) on infestation Day 84; fluralaner efficacy against live-fed *A. americanum* nymphs was > 91% for all 48 h and 72 h counts through infestation Day 56, and was 70.6% (48 h) and 78.0% (72 h) on infestation Day 84 (Table 3).

All live and live-fed *R. sanguineus* (*s.l*.) nymph counts from fluralaner treated dogs at 72 h post-infestation were significantly lower (*P *≤ 0.002) than counts from control dogs for all tick challenge days. Efficacy against all live *R. sanguineus* (*s.l*.) nymphs at 72 h post-infestation was 100% on Day -1, 94% on Day 6, 76.8% on Day 28, 86.3% on Day 56, and 91.9% on Day 84; fluralaner efficacy against live-fed *R. sanguineus* (*s.l*.) nymphs was 100% at all 72 h post-infestation counts for 12 weeks (Table 4).

## Discussion

Although several compounds have been evaluated *in vitro* for efficacy against larval or nymphal stages of ticks [20, 26–30], reports of *in vivo* trials in dogs to determine efficacy against immature stages of ticks are sparse [25]. However, limited field trial data from dogs indicate efficacy [30]; data gleaned from *in vivo* experiments in other animals with other acaricides also indicate efficacy [25, 27]. Oral fluralaner (Bravecto^®^) is labeled for the treatment and control of *A. americanum* for 8 weeks and *R. sanguineus* (*s.l*.) for 12 weeks based on experiments using adult ticks [20, 21]. Although logical to deduce its efficacy against immature tick stages in the field, acaricidal activity of the systemic compound against nymphal *A. americanum* and *R. sanguineus* (*s.l*.) has not been experimentally demonstrated *in vivo* until now. Here, we document efficacy of oral fluralaner in dogs against nymphal *A. americanum* and *R. sanguineus* (*s.l*.) in two separate laboratory controlled experiments. Taken together, efficacy data against all live and live-fed ticks in both experiments demonstrated a single administration of oral fluralaner to dogs at the minimum approved dose was effective against *A. americanum* and *R. sanguineus* (*s.l*.) nymphs for as long as 12 weeks.

Lower than expected (< 90%) fluralaner efficacy against all live nymphs on some tick challenge days in both experiments could be due to several reasons, or combinations thereof. First, the live unengorged nymphs that were not attached to dogs but entrapped in bandage adhesive were enumerated as live, free and not engorged, and were included in counts of all live nymphs used for efficacy calculations. However, these nymphs apparently had not attached to dogs and thus had not ingested the fluralaner. Feeding is necessary for exposure of ticks to isoxazoline acaricides [20]. This is demonstrated by the fact that, with the exception of Day 86 counts for *A. americanum* in the second experiment, fluralaner efficacy was restored to ≥ 90% for both species on all count days when geometric means of live-fed nymphs were analyzed. Additionally, the 12-week efficacy maintained against live-fed *A. americanum* nymphs in the first trial suggests that immature *A. americanum* may be more susceptible to fluralaner than adults, as has been demonstrated for other tick species in *in vitro* experiments [20, 28].

Another explanation for the occasional decrease in fluralaner efficacy against all live counts on some sampling days in both experiments, as well as Day 86 live-fed *A. americanum* counts in the second experiment, is that cohorts of nymphs used for successive infestations may have taken longer to attach and feed. Thus, these nymphs would have ingested less fluralaner before collection at 48 h and 72 h, and the fluralaner would have had less time to exert its acaricidal effects on nymphs prior to their evaluation. Also, in Experiment 2, the 8 dogs transferred from the first experiment may have developed some level of immunity to infestation, which may have decreased efficiency of tick feeding [31] and potential fluralaner ingestion. Still, despite challenges leading to occasional reductions in fluralaner efficacy, overall data indicated that the compound was highly efficacious against *A. americanum* and *R. sanguineus* (*s.l*.) nymphs.

Control of immature ticks is necessary for complete protection of animals from tick infestations. *Amblyomma americanum* are aggressive and feed on a variety of animals during all instars [5]. Because of this generalist feeding behavior, *A. americanum* are exposed to a wide variety of pathogens during all instars and can successfully vector numerous infectious agents to dogs and humans [4, 5, 9]. *Rhipicephalus sanguineus* (*s.l*.), although less diverse in feeding behavior, are well known to establish thriving infestations surrounding and within kennels and homes; infestations with *R. sanguineus* (*s.l*.) instars can be difficult to mitigate once established [4]. If *R. sanguineus* (*s.l*.) are feeding on dogs that are infected with pathogenic agents, this creates a dangerous circumstance for other dogs and humans near or within infested environments [17, 19]. The experimental demonstration of long term *in vivo* acaricidal activity of oral fluralaner against nymphal *A. americanum* and *R. sanguineus* (*s.l*.) indicates the isoxazoline is an appropriate treatment for dogs infested with immature and adult stages of these tick species in the field.

## Conclusions

Empirical evidence from two separate GCP-governed laboratory experiments presented here demonstrates that oral fluralaner (Bravecto^®^) is effective in controlling infestations on dogs with *A. americanum* and *R. sanguineus* (*s.l*.) nymphs for as long as 12 weeks, indicating the compound would be efficacious in the treatment of dogs naturally infested with immature stages of these tick species. Eliminating feeding nymphs and adults on dogs will lead to fewer tick instars developing in the environment that will subsequently go on to feed on new hosts, and thus would be expected to reduce the risk of pathogen transmission.

## Data Availability

The datasets generated and/or analyzed during the present experiments are not publicly available but are available from the corresponding author on reasonable request (upon permission from experiment sponsor).
